# Mg^2+^ Extrusion from Intestinal Epithelia by CNNM Proteins Is Essential for Gonadogenesis via AMPK-TORC1 Signaling in *Caenorhabditis elegans*

**DOI:** 10.1371/journal.pgen.1006276

**Published:** 2016-08-26

**Authors:** Tasuku Ishii, Yosuke Funato, Osamu Hashizume, Daisuke Yamazaki, Yusuke Hirata, Kiyoji Nishiwaki, Nozomu Kono, Hiroyuki Arai, Hiroaki Miki

**Affiliations:** 1 Department of Cellular Regulation, Research Institute for Microbial Diseases, Osaka University, Suita, Osaka, Japan; 2 Laboratory of Health Chemistry, Graduate School of Pharmaceutical Sciences, Tohoku University, Sendai, Miyagi, Japan; 3 Department of Bioscience, School of Science and Technology, Kwansei Gakuin University, Sanda, Hyogo, Japan; 4 Department of Health Chemistry, Graduate School of Pharmaceutical Sciences, The University of Tokyo, Bunkyo-ku, Tokyo, Japan; 5 PRIME, Japan Agency for Medical Research and Development, Chiyoda-ku, Tokyo, Japan; 6 AMED-CREST, Japan Agency for Medical Research and Development, Chiyoda-ku, Tokyo, Japan; Ludwig-Maximilians-Universitat Munchen, GERMANY

## Abstract

Mg^2+^ serves as an essential cofactor for numerous enzymes and its levels are tightly regulated by various Mg^2+^ transporters. Here, we analyzed *Caenorhabditis elegans* strains carrying mutations in genes encoding cyclin M (CNNM) Mg^2+^ transporters. We isolated inactivating mutants for each of the five *Caenorhabditis elegans cnnm* family genes, *cnnm-1* through *cnnm-5*. *cnnm-1*; *cnnm-3* double mutant worms showed various phenotypes, among which the sterile phenotype was rescued by supplementing the media with Mg^2+^. This sterility was caused by a gonadogenesis defect with severely attenuated proliferation of germ cells. Using this gonadogenesis defect as an indicator, we performed genome-wide RNAi screening, to search for genes associated with this phenotype. The results revealed that RNAi-mediated inactivation of several genes restores gonad elongation, including *aak-2*, which encodes the catalytic subunit of AMP-activated protein kinase (AMPK). We then generated triple mutant worms for *cnnm-1*; *cnnm-3*; *aak-2* and confirmed that the *aak-2* mutation also suppressed the defective gonadal elongation in *cnnm-1; cnnm-3* mutant worms. AMPK is activated under low-energy conditions and plays a central role in regulating cellular metabolism to adapt to the energy status of cells. Thus, we provide genetic evidence linking Mg^2+^ homeostasis to energy metabolism via AMPK.

## Author Summary

Mg^2+^ is the second most abundant cation in cells and serves as an essential cofactor for numerous enzymes. To avoid its shortage, cellular and organismal levels of Mg^2+^ are tightly regulated by the concerted actions of various Mg^2+^ transporters and channels. In this study, we analyzed *Caenorhabditis elegans* strains carrying mutations in genes encoding Mg^2+^ transporters and found that the mutations abrogated Mg^2+^ homeostasis. Additionally, these worms were sterile because of a developmental defect in the gonads with severely attenuated proliferation of germ cells. These abnormalities were rescued by additional Mg^2+^ supplementation to the medium, and thus were considered to be due to Mg^2+^ shortage. We investigated the mechanism of this Mg^2+^-associated attenuation of gonadal development, and found that disrupting of the function of AMP-activated protein kinase (AMPK) restored gonad elongation. It is well-known that AMPK is activated under low-energy conditions and plays a central role in regulating cellular metabolism to adapt to the energy status of cells. Thus, we demonstrated that Mg^2+^ homeostasis is intimately connected to energy metabolism via AMPK.

## Introduction

Mg^2+^ is the second most abundant cation in cells and serves as an essential cofactor for numerous enzymes. In mammals, magnesium levels are primarily regulated by intestinal absorption and renal reabsorption, where the epithelial cell layer permits selective and regulated Mg^2+^ transport between apical and basolateral surfaces. There are two known pathways for Mg^2+^ transport through the epithelial cell layers: the paracellular and the transcellular pathways [1]. The transcellular pathway consists of apical entry and basolateral extrusion mediated by Mg^2+^-permeable cation channels and transporters. TRPM6, a member of the transient receptor potential channel (TRP) family, is a key molecule in the transcellular pathway [2]. TRPM6 localizes at the apical membrane of intestinal epithelial cells and distal convoluted tubule (DCT) cells in the kidney [3] and mediates Mg^2+^ absorption and reabsorption, respectively. Indeed, mutations in *TRPM6* result in recessive familial hypomagnesemia with secondary hypocalcemia [4,5]. In addition, the related channel TRPM7 was also found to play an important role in magnesium homeostasis in mice [6]. These observations implicate TRPM6/7 in the apical entry of Mg^2+^ into epithelial cells.

Another key molecule in the transcellular pathway is the ancient conserved domain protein/cyclin M (CNNM) family. In mammals, the CNNM family consists of 4 integral membrane proteins (CNNM1–4) that possess an evolutionarily conserved domain from bacteria [7]. Recent genomic analyses have revealed that several single-nucleotide polymorphisms in *CNNM*s are linked to serum magnesium levels [8] and that mutations in *CNNM2* are responsible for familial hypomagnesemia [9]. It was reported that CNNM4 extrudes Mg^2+^ from the basolateral membrane of intestinal epithelial cells and is involved in intestinal Mg^2+^ absorption [10]. Another family member, CNNM2, is strongly expressed at the basolateral membrane of DCT cells [9,11] and can extrude Mg^2+^ similarly to CNNM4 [12], suggesting that CNNM2 plays a similar role in basolateral Mg^2+^ extrusion in kidney DCT cells.

Two groups recently reported that CNNMs associate with phosphatase of regenerating liver (PRL), a cancer-associated tyrosine phosphatase [13,14]. One group found that PRL binds to CNNMs and inhibits the Mg^2+^-transporting function [13], whereas the other group reported that PRL stimulates this activity [14]. Therefore, how PRL affects the function of CNNMs remains unclear. In addition, it is unknown whether other molecules are involved in the regulation of CNNM function. To address these problems, comprehensive screening can be used to identify genes that functionally associate with CNNMs. *Caenorhabditis elegans* (*C*. *elegans*), which is a model organism commonly used for genetic analyses, also absorbs Mg^2+^ through a similar transcellular mechanism in the intestine. The apical entry step of the transcellular pathway is mediated by two TRPM family channels: GTL-1 and GON-2 [15]. *C*. *elegans* has an excretory canal that removes wastes from the body, wherein another *C*. *elegans* TRPM channel, GTL-2, plays an important role in magnesium homeostasis [16]. Thus, *C*. *elegans* possesses a system for regulating magnesium homeostasis that is similar to that in mammals. Taken together with the genetic tractability of *C*. *elegans*, this organism may serve as an ideal experimental model for investigating the regulatory mechanism and functional importance of magnesium homeostasis.

In this study, we performed functional analyses of the *C*. *elegans* CNNM family and found that *cnnm-1*; *cnnm-3* double mutant worms displayed pleiotropic phenotypes. The sterile phenotype (due to defective gonadogenesis) was rescued by Mg^2+^ supplementation: oocyte development was restored and mutant worms became fertile. Detailed analyses of the gonadal phenotype revealed that the inactivating mutation of *aak-2*, which encodes the α subunit of AMP-activated protein kinase (AMPK), significantly rescued the gonadogenesis defect in *cnnm-1*; *cnnm-3* mutants, thereby indicating a genetic interaction between CNNM and AMPK.

## Results

### *cnnm* family genes of *C*. *elegans*

**Fig 1:**
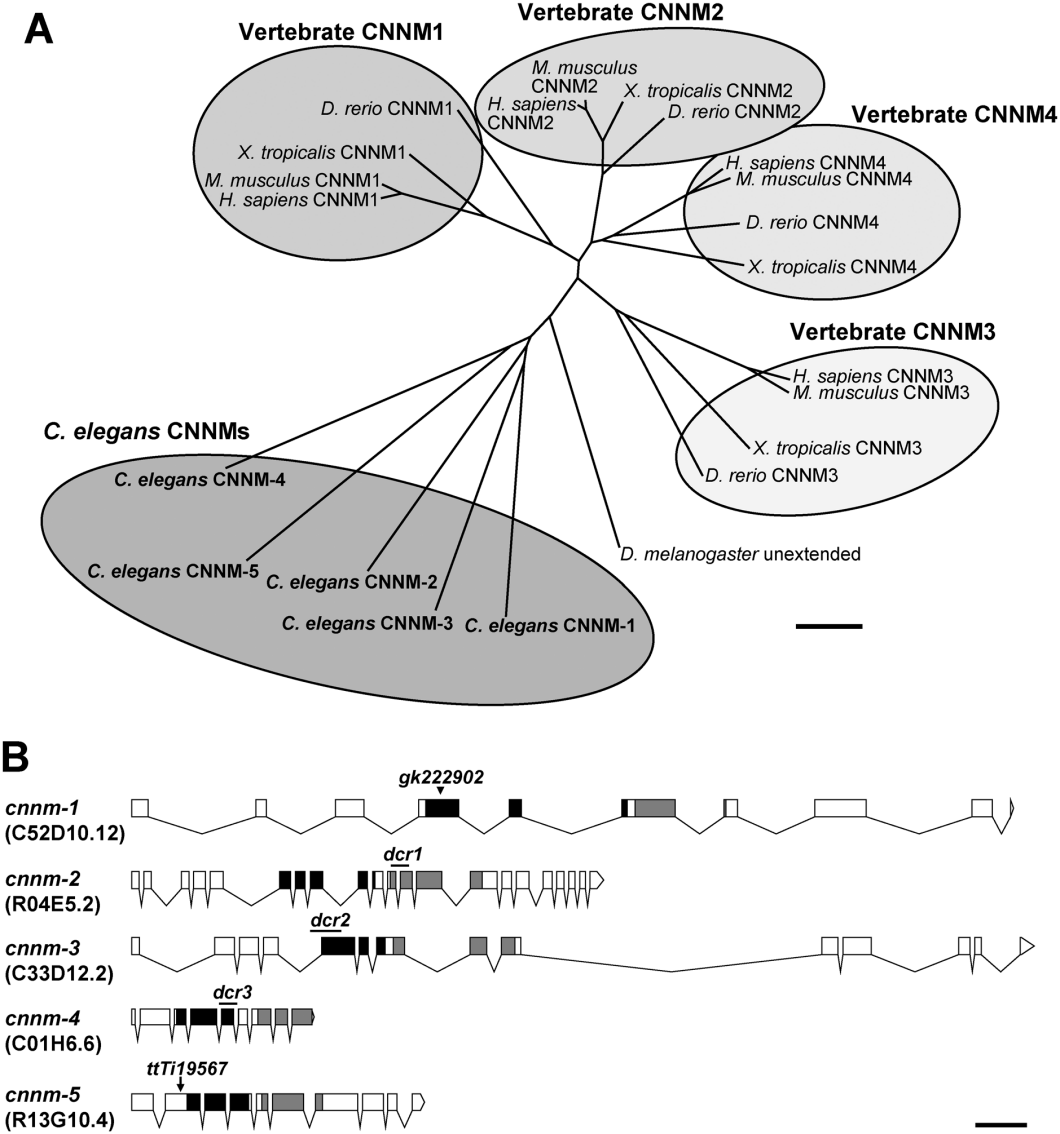
The *C*. *elegans cnnm* family. (A) Phylogenetic tree of CNNM family. Amino acid sequences of *C*. *elegans* CNNMs, along with human, mouse, frog, zebrafish, and fruit fly CNNM orthologues, were aligned using Clustal W (version 2.1, http://clustalw.ddbj.nig.ac.jp/), and the phylogenetic tree was generated with neighbor-joining method by using Clustal W2—phylogeny (http://www.ebi.ac.uk/Tools/phylogeny/clustalw2_phylogeny/). Vertebrate CNNM orthologues are grouped, and *C*. *elegans* CNNMs are also grouped. Bar, 0.1 substitutions per amino acid. (B) Schematic illustrations of the *cnnm* family genes. Exons and introns are indicated by boxes and lines, respectively. The regions encoding the evolutionarily conserved DUF21 and CBS domains are indicated with black and gray boxes, respectively. Sequence names are shown in parentheses. The illustrations were generated using the Exon-Intron Graphic Maker by Nikhil Bhatla (http://www.wormweb.org/exonintron). Lines, deletion; arrowhead, point mutation; arrow, *Mos1* insertion. Bar, 0.5 kb.

A homology search using BLAST with amino acid sequences of the human CNNM4 protein revealed that the *C*. *elegans* genome contains genes encoding 5 previously uncharacterized CNNM family proteins, which possess the functionally essential domains DUF21 and CBS [10,12] (S1 Fig). Each *C*. *elegans* CNNM protein showed significant identity with all human CNNM family members (24–47%). To determine the evolutionary relationship between *C*. *elegans* CNNM proteins and other CNNM homologs, we constructed a phylogenetic tree (Fig 1A). The vertebrates have 4 paralogs (CNNM1–4), and each of them is orthologous between different vertebrate species (human, mouse, frog, and zebrafish). In contrast, *C*. *elegans* CNNM-1−5 emerged independently of the vertebrate CNNMs. To investigate the *in vivo* functions of *C*. *elegans* CNNM family proteins, we obtained and generated mutant alleles for all *cnnm* family members (Fig 1B). These mutations abolish the function of each CNNM protein because the mutant proteins lack functionally essential part of either the DUF21 or CBS domains (S1 Fig, see its legend for details).

### Pleiotropic phenotypes of *cnnm-1; cnnm-3* mutant worms

**Fig 2:**
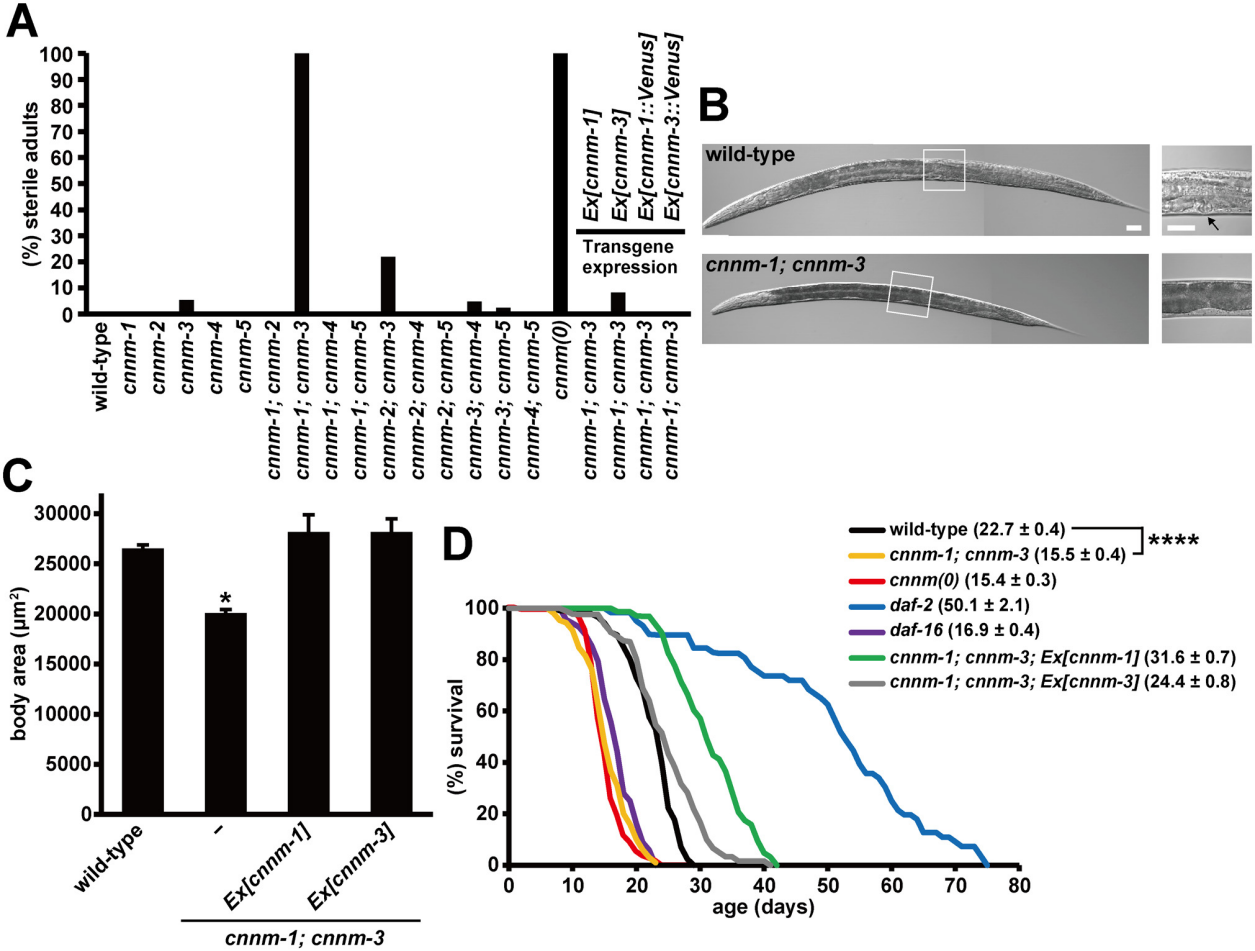
Pleiotropic phenotypes of *cnnm-1; cnnm-3* mutant worms. (A) Quantification of the sterile phenotype for each genotype. Eggs collected by synchronous laying were grown for 4 days and then examined for the presence of embryos in their uteri by microscopic observation. Worms lacking embryos were scored as sterile. More than 100 worms were analyzed for each genotype. *cnnm(0)*: *cnnm-1*; *cnnm-2*; *cnnm-3*; *cnnm-4*; *cnnm-5* mutant. (B) Nomarski images of wild-type and *cnnm-1*; *cnnm-3* mutant worms grown for 3 days. High-magnification views of the boxed areas are also shown (right). Arrow indicates the vulva. Bar, 30 μm. (C) Mixed stage worms from L2 to adult (L4 to adult worms were most abundant) were examined for alae formation and subjected to body size measurement (*n* = 30 in each experiment). The body size at the transition from L4 to the adult molt was estimated by calculating the mean body size of the smallest three worms with alae and the largest three worms without alae. The data are shown as the means of three experiments. Error bars indicate SEM. *p* values were determined by ANOVA, followed by two-tailed multiple Student’s *t*-test with Tukey’s correction. *p < 0.05 versus wild-type. (D) Lifespan of worms with the indicated genotype. For each genotype, 45 synchronized L4/young adult worms were transferred to fresh plates (15 worms per plate) and were then scored daily for survival. The graph represents data combined from at least two experiments. Mean lifespan (± SEM) of worms is also indicated in parentheses. *p* values were determined by log rank (Mantel-Cox) test, and the Bonferroni method was then used to correct for multiple comparisons. ****p < 0.0001.

Observation of these mutant worms revealed no obvious abnormalities except for in the *cnnm-3* mutant worms, a few (5.3%) of which were sterile (Fig 2A). We speculated that functional redundancy among the *cnnm* family members may have masked the mutant phenotype. Thus, we generated double mutants for all possible combinations by crossing all single mutants. We found that *cnnm-1*; *cnnm-3* and *cnnm-2*; *cnnm-3* mutant worms were severely (100%) and moderately (22%) sterile, respectively (Fig 2A). Because of the completely sterile phenotype, we analyzed *cnnm-1*; *cnnm-3* mutants in subsequent experiments.

The *cnnm-1*; *cnnm-3* mutant worms were significantly smaller than the wild-type N2 worms grown for the same amount of time (Fig 2B). Therefore, we compared the body sizes of stage-matched worms. *cnnm-1*; *cnnm-3* mutant worms did not form the vulva (Fig 2B), the eversion of which determines the adult stage [17]. Therefore, we focused on the presence of alae, the longitudinal ridges present in adult worms but not in earlier L2−4 larvae as the marker to confirm whether the worms had reached the adult stage [18,19]. We examined alae formation and body sizes of mixed stage worms from L2 to adult, and then estimated the body size at the transition from L4 to adult molt, which was determined as the mean value of body size of the three smallest worms with alae and three largest worms without alae. The results showed that the body size of *cnnm-1*; *cnnm-3* mutant worms was smaller than that of wild-type worms (Fig 2C). At 64 h when all wild-type worms had just reached the adult stage, approximately half of the *cnnm-1*; *cnnm-3* mutant worms were alae-positive, indicating developmental delay. These phenotypes of *cnnm-1*; *cnnm-3* mutant worms were rescued by the introduction of either *cnnm-1* or *cnnm-3* genomic DNA (Fig 2A and 2C), confirming that these abnormalities were caused by mutations in *cnnm-1* and *cnnm-3*.

We also found that the color of the intestine in *cnnm-1*; *cnnm-3* mutant worms was dark (Fig 2B). This was also observed in mutant worms of *daf-2*, which encodes the insulin-like receptor [20]. Because *daf-2* mutations are well-known to extend lifespan, we next examined the lifespan of *cnnm-1*; *cnnm-3* mutant worms (Fig 2D). Consistent with previous studies, *daf-2* mutant worms showed much longer lifespans than wild-type worms. In contrast, *cnnm-1*; *cnnm-3* mutant worms had shorter lifespans than wild-type worms, which were similar to those of *daf-16* mutant worms; these worms were used here as an example of short-lived mutants [20]. Introduction of either *cnnm-1* or *cnnm-3* genomic DNA to *cnnm-1*; *cnnm-3* mutant worms not only rescued the short-life phenotype, but also showed moderately longer lifespans than wild-type worms, particularly in the case of worms introduced with *cnnm-1* (Fig 2D). Overexpression of either *cnnm-1* or *cnnm-3* in wild-type worms also prolonged the lifespan (S2 Fig), indicating the important role of these genes in determining lifespan.

We also analyzed whether the short-life phenotype of *cnnm-1*; *cnnm-3* mutants could be exacerbated by further mutations in other CNNM family genes. For this, we generated *cnnm-1*; *cnnm-2*; *cnnm-3*; *cnnm-4*; *cnnm-5* quintuple mutant worms (hereafter, *cnnm(0)*), which possessed mutations in all five *C*. *elegans cnnm* family genes, by mating. As expected, *cnnm(0)* worms showed complete sterility (Fig 2A) with a lifespan similar to that of *cnnm-1*; *cnnm-3* mutants (Fig 2D).

### Effects of Mg^2+^ supplementation on *cnnm-1; cnnm-3* mutant worms

**Fig 3:**
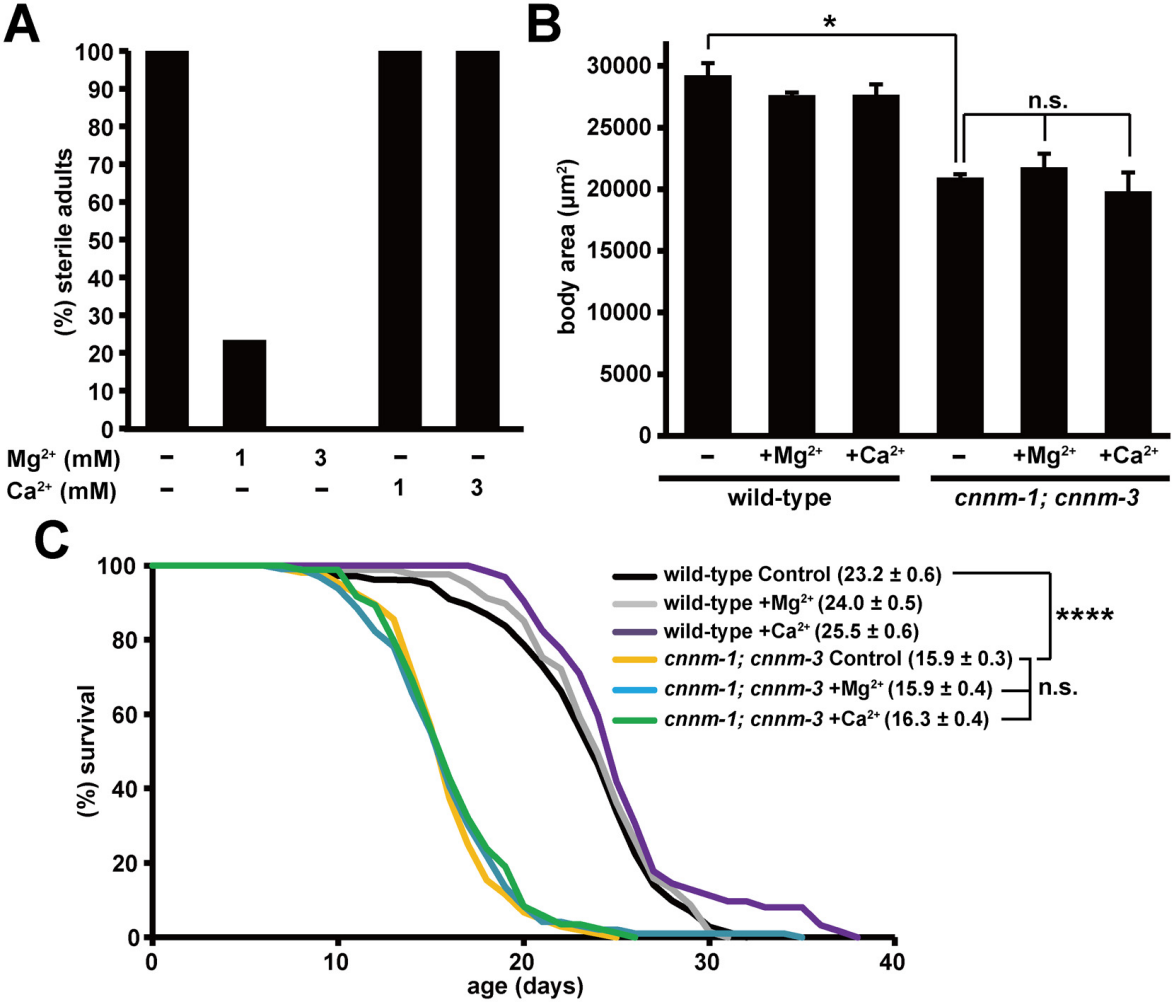
Effects of Mg^2+^ supplementation on *cnnm-1*; *cnnm-3* mutant worms. (A) *cnnm-1*; *cnnm-3* mutant worms were grown on plates supplemented with the indicated concentrations of Mg^2+^ or Ca^2+^ for 4 days and then examined for sterility. More than 50 worms were analyzed for each experimental condition. (B) Wild-type and *cnnm-1*; *cnnm-3* mutant worms were grown on plates supplemented with 1 mM Mg^2+^ or Ca^2+^ for at least two generations. Mixed stage worms from L2 to adult (L4 to adult worms comprise the most) were examined for alae formation and subjected to body size measurement (*n* = 30 per each experiment). Body size at the transition from L4 to adult molt was estimated as in Fig 2C. The data are shown as the means of three experiments. Error bars indicate SEM. *p* values were determined by ANOVA, followed by two-tailed multiple Student’s *t*-tests with Tukey’s correction. *p < 0.05. (C) 45 synchronized L4/young adult worms of the indicated genotype were transferred to fresh plates supplemented with 1 mM Mg^2+^ or Ca^2+^ (15 worms per plate), and then scored for survival daily. The graph represents data combined from at least two experiments. Mean lifespan (± SEM) of worms is also indicated in parentheses. *p* values were determined by log rank (Mantel-Cox) test, and the Bonferroni method was then used to correct for multiple comparisons. ****p < 0.0001.

Because mammalian CNNM family proteins are involved in Mg^2+^ transport [10,12–14], the phenotypes of *cnnm-1*; *cnnm-3* mutant worms may be related to abnormalities in magnesium homeostasis. Therefore, we tested the effects of Mg^2+^ supplementation in the media, and found that 76.6% and 100% of *cnnm-1*; *cnnm-3* mutant worms became fertile following supplementation with 1 mM and 3 mM of Mg^2+^, respectively (Fig 3A). In contrast, the small body size and short lifespan were not affected by Mg^2+^ supplementation (Fig 3B and 3C). Supplementation of culture plates with Ca^2+^ did not affect any of the phenotypes. Collectively, these results suggest that the sterile phenotype of *cnnm-1*; *cnnm-3* mutant worms is related to altered magnesium homeostasis. As the *cnnm-1*; *cnnm-3* mutant worms did not produce oocytes or form a vulva (Fig 2B), which are formed during gonadal development, we next examined gonadal development in *cnnm-1*; *cnnm-3* mutant worms.

### *cnnm-1* and *cnnm-3* are required for postembryonic gonadal development

**Fig 4:**
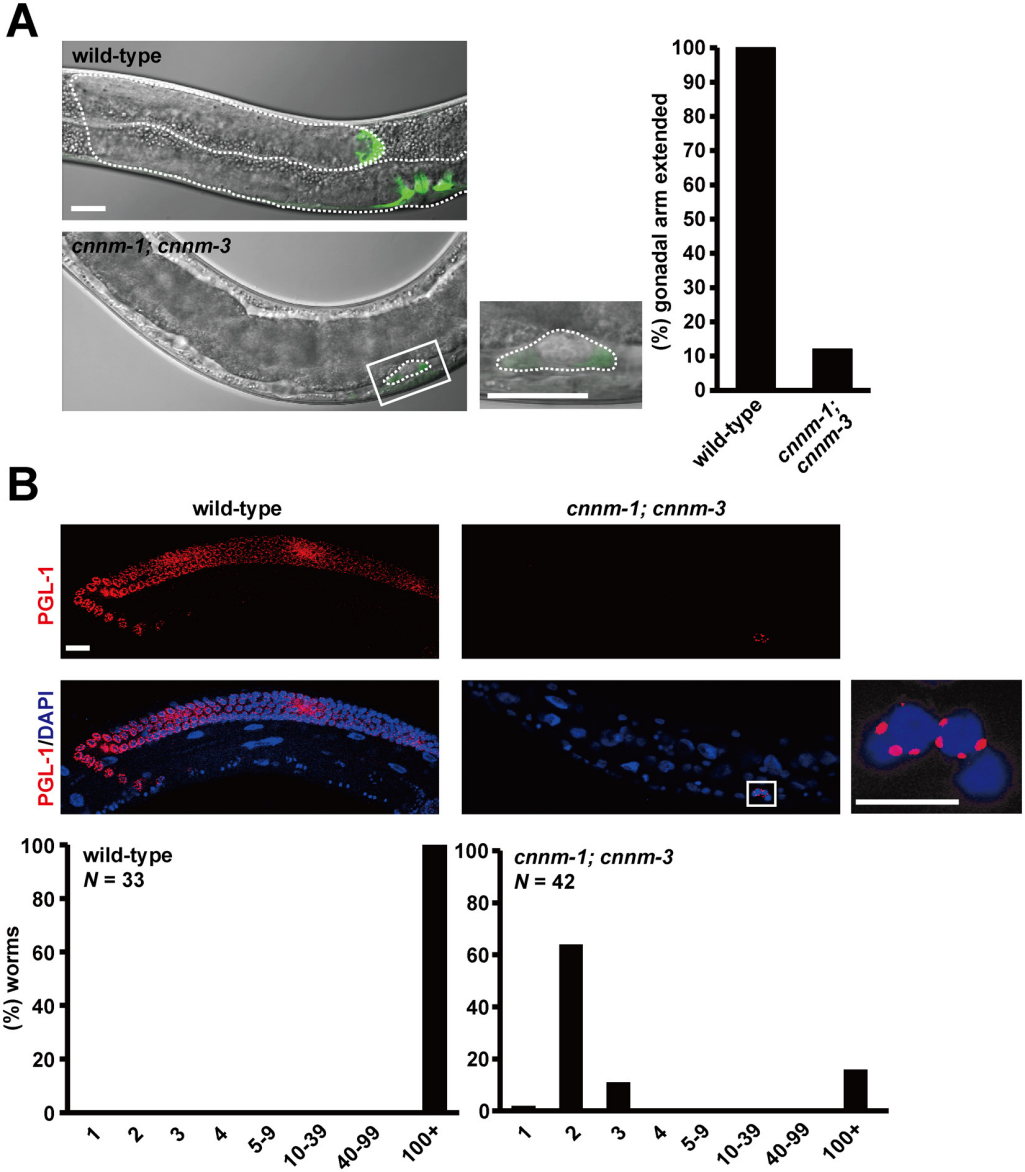
*cnnm-1* and *cnnm-3* are required for postembryonic development of the gonad. (A) L4/young adult worms of the indicated genotype, carrying *lag-2p*::*GFP*, were examined for gonadal arm extension. Representative images and the high-magnification view of the boxed area are shown (left). In each image, the anterior side of the worm is positioned to the left. Dotted lines indicate the outlines of the gonad. Bar, 20 μm. Worms with gonads of ≥ 60 μm in length of the long axis (3-fold as long as the primordial gonad) were determined as extended and the results (%) are shown on the graph (right). More than 100 worms were analyzed. (B) The worms were stained with DAPI (blue) and anti-PGL-1 antibody (red), and then the number of germ cells (PGL-1-positive cells) per worm was counted. Representative images and the high-magnification view of the boxed area are shown (top). Bar, 15 μm. The graph indicates the distribution of worms (%) with the indicated numbers of germ cells (bottom).

At hatching, the primordial gonad in *C*. *elegans* is composed of four cells: Z1–Z4 [21]. During larval development, Z1 and Z4 cells give rise to the somatic gonad including the distal tip cells (DTCs), uterus, sheath cells, and spermathecae, whereas the Z2 and Z3 cells give rise to the germ line. We examined the extent of gonadal development by expressing GFP under control of the *lag-2* promoter, which drives gene expression in Z1/Z4 cells and DTCs [22,23] located at the distal end of two gonadal arms and lead the elongation of the arms to form the U-shaped hermaphrodite gonad. When the mutant worms hatched from eggs, two GFP-positive cells were observed at the appropriate positions, suggesting that primordial gonad development proceeded normally in mutants. However, the primordial gonad of mutants did not elongate, even in the L4/young adult stages (Fig 4A). Immunofluorescence analysis using an anti-PGL-1 antibody, which stains P-granules in germ cells, demonstrated that most of the *cnnm-1*; *cnnm-3* mutant L4/young adult worms had only two germ cells corresponding to the Z2/Z3 cells (Fig 4B). Considering that Mg^2+^ supplementation restored the fertility of *cnnm-1; cnnm-3* mutant worms, these results suggest that CNNM-1 and CNNM-3 promote postembryonic gonadal development by regulating Mg^2+^ levels. Previous studies demonstrated that mutants of *gon-2*, which encodes a TRPM channel protein, showed a severe gonadogenesis defect, which was partially restored by Mg^2+^ supplementation [15,24].

In addition to somatic gonad and germ cells, several types of cells, such as the ray cells of the male tail, are known to proliferate during postembryonic stages [25]. Our findings revealed no apparent abnormalities in the tail ray structures of *cnnm-1*; *cnnm-3* mutant males (S3 Fig), suggesting that not all of the postembryonic cell divisions were affected in *cnnm-1; cnnm-3* mutant worms.

### Localization of CNNM-1 and CNNM-3 at the basolateral membrane of intestinal cells

**Fig 5:**
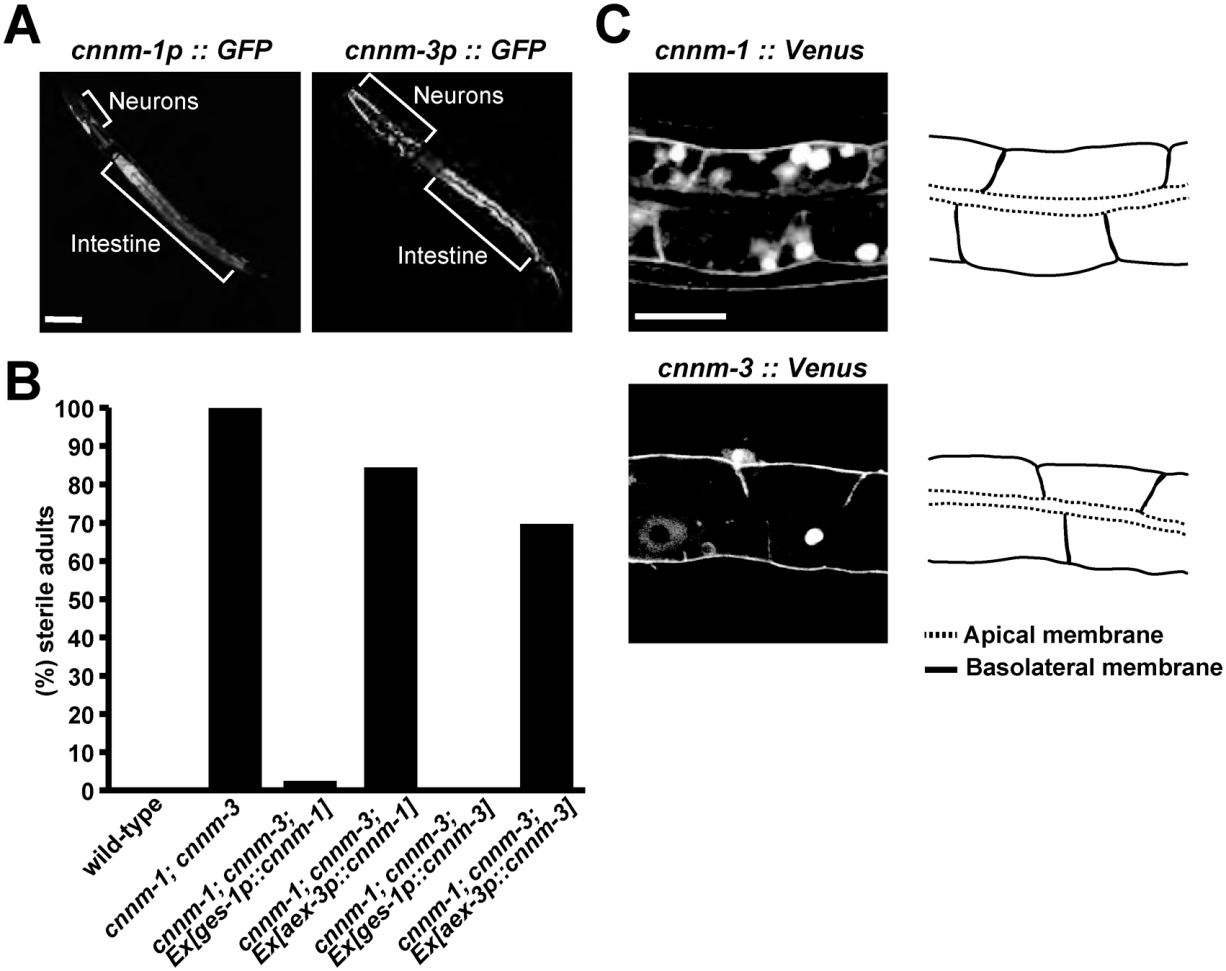
Localization of CNNM-1 and CNNM-3 at the basolateral membrane of intestinal cells. (A) Fluorescent images of wild-type L1 larvae with *cnnm-1p*::*GFP* or *cnnm-3p*::*GFP*. Areas of neurons and intestines showing strong GFP signals are marked. Bar, 30 μm. (B) Quantification of sterile phenotype in wild-type, *cnnm-1*; *cnnm-3* mutants, and *cnnm-1*; *cnnm-3* mutants expressing either CNNM-1 or CNNM-3 under the control of the promoters for intestinal (*ges-1p*) or pan-neuronal (*aex-3p*) markers. More than 50 worms were analyzed for each genotype. (C) Fluorescent images of intestinal cells from wild-type L1 larvae expressing the Venus-fusion proteins of CNNM-1 or CNNM-3 (left). Schematic representation of the intestinal cell plasma membranes are also shown (right). Apical and basolateral membranes are indicated by dotted and solid lines, respectively. Bar, 10 μm.

To characterize the roles of CNNM-1 and CNNM-3, we first examined their expression patterns by generating transgenic worms expressing GFP under control of the *cnnm-1* or *cnnm-3* promoters. Unique GFP expression was observed in various tissues, such as the pharynx, hypodermis, rectum, and muscles, but strong expression was commonly observed in the intestine and neurons (Fig 5A). Given this expression pattern, we forced the expression of CNNM-1 or CNNM-3 in the intestine or neurons of *cnnm-1*; *cnnm-3* mutant worms using the intestine-specific *ges-1* promoter [26] or neuron-specific *aex-3* promoter [27], respectively. The intestinal expression of either CNNM-1 or CNNM-3 nearly completely rescued the sterile phenotype of *cnnm-1*; *cnnm-3* mutants, whereas their expression in neurons showed only a subtle effect (Fig 5B), suggesting that their expression in the intestine is important for gonadal development. The intestinal cells of *C*. *elegans* are attached to each other at the borders of the apical membrane via cell-cell junctions known as apical junctions, which have mixed traits of both the adherens junction and the tight junction in mammalian epithelial cells, and thus exhibit apico-basal polarity [28]. We subsequently examined the subcellular localization of CNNM-1 and CNNM-3 in intestinal cells using transgenic worms expressing their respective Venus-fusion proteins, which also rescued the sterile phenotype (Fig 2A). Excluding some large clumps in the cytoplasm, which are often observed following ectopic expression of Venus-fusion proteins, the fluorescent signal of Venus-fusion CNNM1 and CNNM3 was predominantly observed in the basolateral membrane of intestinal cells (Fig 5C). Therefore, both CNNM-1 and CNNM-3 are considered to extrude Mg^2+^ from intestinal cells to the pseudocoelom. Based on this hypothesis, *cnnm-1*; *cnnm-3* mutant worms were expected to have higher levels of Mg^2+^ in intestinal cells and lower levels of Mg^2+^ in the pseudocoelom, which contains other tissues such as the gonad, possibly explaining why Mg^2+^ supplementation restored gonadal development (Fig 3A).

### Assessment of magnesium levels by ICP-MS

To assess the predicted regulatory model, we quantified the magnesium levels in wild-type worms and in *cnnm-1*; *cnnm-3* mutant worms using inductively coupled plasma mass spectrometry (ICP-MS). As shown in Table 1, we found that *cnnm-1*; *cnnm-3* mutant worms had higher magnesium levels (143% of wild-type worms). We then physically dissected the intestines from the worms using a scalpel and subjected the intestines to magnesium quantitation. The results indicated much higher levels of magnesium in *cnnm-1; cnnm-3* mutant worms (195% of wild-type worms). It should be noted that there was no overlap in magnesium levels in wild-type and mutant worms. It was technically difficult to physically dissect other remaining tissues, which are much smaller than the intestine. Therefore, we estimated magnesium levels in other tissues by calculating the volumes of the total body and intestine, and found that the magnesium level was reduced in other tissues of *cnnm-1*; *cnnm-3* mutant worms (67% of wild-type levels). Taken together with the result that the infertile phenotype was rescued by artificially increasing Mg^2+^ levels (Fig 3), the abnormalities in *cnnm-1*; *cnnm-3* mutant worms may have been caused by Mg^2+^ deficiency.

**Table 1:** Magnesium levels in wild-type and *cnnm-1; cnnm-3* mutant worms.

	wild-type	*cnnm-1; cnnm-3*	*p*-value
**Total (ppb/μg)**	3.9 ± 0.2	5.6 ± 0.4	0.014
**Intestine (ppb/μg)**	4.0 ± 0.4	7.8 ± 0.8	0.015
**Other tissues (ppb/μg) (estimated value)**	3.9	2.6	ND

The total and intestinal levels of magnesium were directly determined by ICP-MS using L4/young adult worms. The data are shown as the means (± SEM) of three experiments. Magnesium levels in the other remaining tissues were estimated by calculating the total body volume (wild-type: 1,170 ± 61 nl, *n* = 10; *cnnm-1; cnnm-3*: 638 ± 38 nl, *n* = 10) and intestine (wild-type: 458 ± 22 nl, *n* = 10; *cnnm-1; cnnm-3*: 357 ± 13 nl, *n* = 10). *p* values were determined by Student’s two tailed *t*-test.

### RNAi screening for genes that functionally associated with *cnnm-1* and *cnnm-3*

Next, we searched for genes that are functionally associated with *cnnm-1* and *cnnm-3*. For this purpose, we performed RNAi-based screening, because (i) an RNAi feeding library targeting 86% of the open reading frames of *C*. *elegans* is available and widely used in genome-wide screening [29] and (ii) unlike conventional forward genetics approaches, it is not necessary to collect the worms and their progenies after screening, making this method applicable to sterile *cnnm-1*; *cnnm-3* strains. To increase RNAi efficacy, screening was performed using worms carrying the *rrf-3* mutation, which renders the worms hypersensitive to RNAi treatment [30]. Two rounds of screening identified 31 genes, of which RNAi treatment reproducibly resulted in elongation of the gonadal arm in more than 50% of *cnnm-1*; *cnnm-3*; *rrf-3* mutant worms (Table 2 and S4 Fig). These 31 genes are involved in a variety of biological processes, including protein transport, metabolism, mitochondrial function, signal transduction, gene expression, ion transport, immune response, and the cell cycle. Among these, we performed detailed analyses of *aak-2*, which encodes the α-subunit of AMPK. AMPK is the key energy sensor in most eukaryotic cells and is activated under low-energy conditions such as decreased ATP levels [31]. Most intracellular ATP is known to form complexes with Mg^2+^, which is required for numerous enzymatic reactions involving ATP [32,33]. We hypothesized that dysregulation of cellular Mg^2+^ levels in *cnnm-1*; *cnnm-3* mutant worms could affect AMPK activity.

**Table 2:** Genes of which RNAi suppressed the gonadogenesis defect in *cnnm-1; cnnm-3* mutant worms.

			(%) gonadal arm extended	
Sequence name	Gene name	Description	1st round	2nd round
C10E2.6	*mct-6*	Monocarboxylate transporter	100	100
R160.1	*dpy-23*	AP-2 complex subunit mu2	100	100
C06B8.7		Scavenger receptor cysteine-rich domain	100	100
F55D10.3	*glit-1*	Thyroglobulin	80	93
T05H4.5	*hpo-19*	NADH-cytochrome b5 reductase	90	90
C55B7.8	*dbr-1*	RNA-lariat debranching enzyme	90	87
F02E8.3	*aps-2*	AP-2 complex subunit sigma2	90	87
T01C8.1	*aak-2*	AMPK alpha2	80	87
C34C6.6	*prx-5*	Peroxisomal targeting signal 1 receptor	80	87
R03E1.1	*sym-4*	WD repeat-containing protein	80	83
F29A7.6		M-phase phosphoprotein 6	100	83
T14G10.7	*hpo-5*	GPI transamidase component PIG-S	70	83
C07A9.11	*ncx-7*	Sodium/potassium/calcium exchanger	90	80
R12C12.2	*ran-5*	RanBP1 domain	80	77
F11E6.5	*elo-2*	palmitic acid elongase	50	73
F36H12.5		Transcription initiation factor TFIID subunit 3	80	73
F44A6.2	*sex-1*	Nuclear hormone receptor	60	73
F58B3.4		Nucleolar pre-rRNA processing protein	80	70
C27F2.8		Transmembrane protein 131	80	67
T26A8.4		Zinc finger CCCH domain-containing protein 4	80	67
K04E7.2	*pept-1*	Oligopeptide transporter	80	67
Y65B4BR.4	*wwp-1*	NEDD4-like E3 ubiquitin-protein ligase WWP1	50	63
C09H6.3	*mau-2*	MAU2 chromatid cohesion factor homolog	70	60
C27B7.8	*rap-1*	Ras-related protein Rap-1b	50	60
F23C8.6	*did-2*	Charged multivesicular body protein 1b	50	57
F08B12.2	*prx-12*	Peroxisomal biogenesis factor 12	60	57
ZK418.4	*lin-37*	LIN37 family protein	70	53
R07H5.8		Adenosine kinase	60	53
Y71H10B.1		Cytosolic purine 5'-nucleotidase	50	53
Y40B1B.7		Coiled-coil domain-containing protein 86	50	53
F46F11.4	*ubl-5*	Ubiquitin-like protein 5	50	50

Genome wide RNAi screening identified 31 genes that functionally associate with *cnnm-1* and *cnnm-3*. The information is derived from either Wormbase or InterProScan.

### AMPK mediates the gonadogenesis defect caused by *cnnm-1*; *cnnm-3* mutation

**Fig 6:**
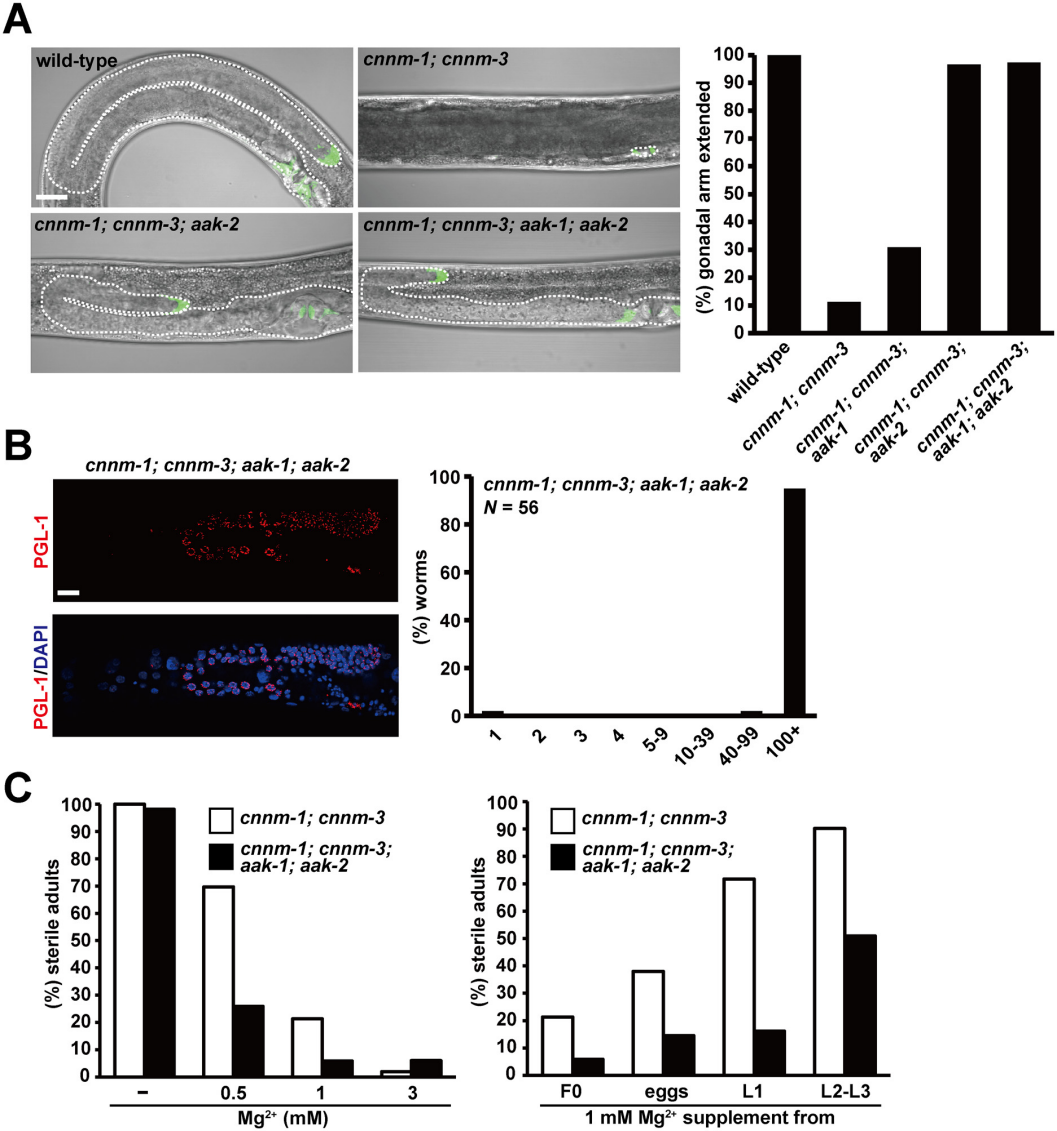
AMPK mediates the gonadogenesis defect of *cnnm-1*; *cnnm-3* mutant worms. (A) L4/young adult worms of the indicated genotype, carrying *lag-2p*::*GFP*, were examined for gonadal arm extension. Representative images are shown (left). In each image, the anterior side of the worm is positioned to the left. Dotted lines indicate the outlines of the gonad. Bar, 20 μm. Worms with gonads of ≥ 60 μm in length of the long axis (3-fold as long as the primordial gonad) were determined as extended and the results (%) are shown in the graph (right). More than 100 worms were analyzed. (B) *cnnm-1*; *cnnm-3*; *aak-1*; *aak-2* mutant worms were stained with DAPI (blue) and anti-PGL-1 antibody (red), and then the number of germ cells (PGL-1-positive cells) per worm was counted. Representative images are shown (left). Bar, 15 μm. The graph indicates the distribution of worms (%) with the indicated numbers of germ cells (right). (C) Worms of the indicated genotype were grown on plates supplemented with the indicated concentrations of Mg^2+^ from the F0 generation (left) or grown on plates supplemented with 1 mM Mg^2+^ starting from the indicated stages (right). More than 50 worms were analyzed for each experimental condition.

AMPK is a heterotrimeric kinase consisting of a catalytic subunit (α) and two regulatory subunits (β and γ). In *C*. *elegans*, there are two α subunits, AAK-1 and AAK-2, which are encoded by different genes [34]. Therefore, we examined whether the predicted null mutations for *aak-1* and/or *aak-2* could suppress the gonadogenesis defect in *cnnm-1*; *cnnm-3* mutant worms (Fig 6A). The additional mutation in *aak-2* or in both *aak-1* and *aak-2* nearly completely restored the gonadal arm extension, while the *aak-1* mutation showed only a marginal effect. We also analyzed germ cell proliferation in *cnnm-1*; *cnnm-3*; *aak-1*; *aak-2* quadruple mutant worms and found that most contained many germ cells (> 100 cells, Fig 6B). Moreover, when we examined the fertility of quadruple mutant worms by supplementation with various concentrations of Mg^2+^, fertility was restored at lower concentrations of Mg^2+^ (Fig 6C, left). We also performed rescue experiments by changing the initiation timing of Mg^2+^ supplementation. The results showed that fertility was restored even when supplementation was started at later stages (Fig 6C, right). Therefore, the additional mutations in *aak-1* and *aak-2* significantly augmented the effect of Mg^2+^ on fertility, implicating that AMPK mediates the effect of Mg^2+^ on gonadogenesis.

**Fig 7:**
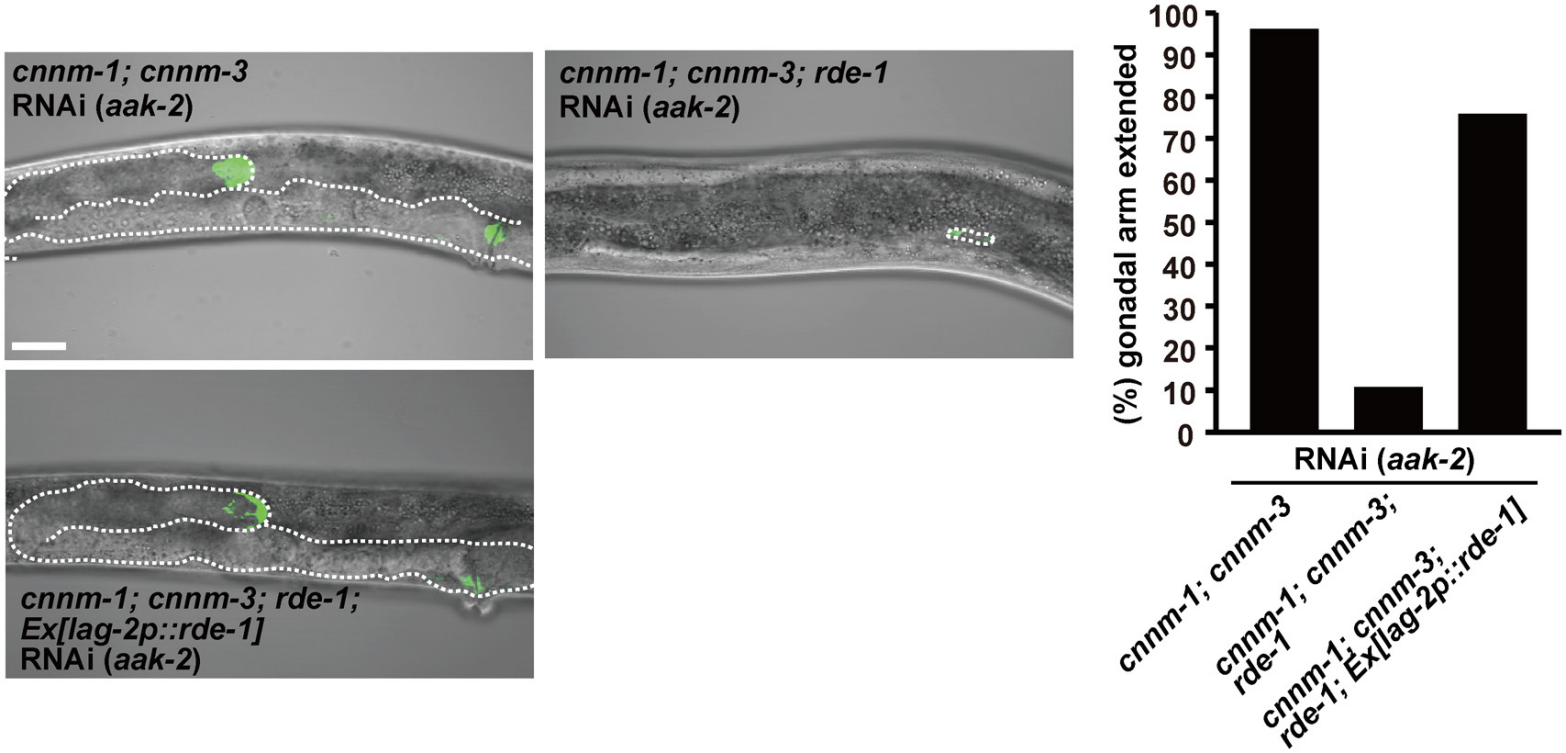
Primary defect occurs in somatic gonad. L4/young adult worms of the indicated genotype, carrying *lag-2p*::*GFP*, were fed with bacteria carrying the dsRNA corresponding to *aak-2*. In each image, the anterior side of the worm is positioned to the left. Dotted lines indicate the outlines of the gonad. Bar, 20 μm. Worms with gonads of ≥ 60 μm in length of the long axis (3-fold as long as the primordial gonad) were determined as extended and the results (%) are shown in the graph (right). More than 50 worms were analyzed for each condition.

To identify the cell type that is primarily affected by the *cnnm-1*; *cnnm-3* mutations and responsible for the gonadogenesis defect, we performed tissue-specific RNAi experiments. It has been reported that somatic gonad-specific RNAi can be achieved using a strain that carries both a mutation in the *rde-1* gene, which encodes an Argonaute protein required for siRNA maturation [35], and a transgene *qIs140[lag-2p*::*rde-1]* that drives the expression of wild-type *rde-1* under the control of the *lag-2* promoter [36]. We generated *cnnm-1*; *cnnm-3*; *rde-1*; *qIs140* strains and performed feeding RNAi experiments of *aak-2*. As shown in Fig 7, somatic gonad-specific RNAi of *aak-2* restored the gonadal arm extension in most (75.9% extended) *cnnm-1*; *cnnm-3*; *rde-1*; *qIs140* worms, while worms without the transgene (*cnnm-1*; *cnnm-3*; *rde-1*) failed to show restored extension. These results clearly show the importance of *aak-2* function in the somatic gonad.

AMPK can regulate multiple downstream molecules, including the target of rapamycin complex (TORC)1, which is well-known to be important in cell growth [37]. Therefore, we performed RNAi experiments to investigate the importance of TORC1 in gonadogenesis. Because RNAi of either *let-363* or *daf-15* is known to cause L3 larval arrest [38], we observed worms in the L2–L3 stages. We found that inhibition of *daf-15* (encoding Raptor ortholog) or *let-363* (encoding ortholog of mammalian TOR kinase) efficiently suppressed gonadal arm extension in *cnnm-1*; *cnnm-3*; *aak-1*; *aak-2* quadruple mutant worms (S5 Fig). In contrast, inhibition of *rheb-1* (encoding Rheb ortholog) showed minimal suppression of gonadal arm extension. Biochemical analyses using mammalian cultured cells showed that AMPK inhibited TOR kinase via phosphorylation of TSC (the upstream regulator of Rheb [39]) and Raptor [40], but there is no TSC homolog in *C*. *elegans* [41]. Taken together, the results of our RNAi experiments suggest the involvement of dysregulated AMPK-TORC1 signaling in the gonadogenesis defect of *cnnm-1*; *cnnm-3* mutants.

## Discussion

In this study, we showed that *cnnm-1*; *cnnm-3* mutant worms displayed pleiotropic phenotypes, such as infertility due to a gonadogenesis defect, shortened lifespan, and small body size (Fig 2). Among these, the gonadogenesis defect was completely restored by adding Mg^2+^ to the culture media (Fig 3). These results suggest that abnormal Mg^2+^ regulation in *cnnm-1*; *cnnm-3* mutant worms affected gonadal development. In contrast, Mg^2+^ supplementation affected neither the lifespan nor the body size of *cnnm-1*; *cnnm-3* mutants (Fig 3). Whether the lifespan and body size phenotypes are related to altered magnesium homeostasis remains unknown, and further analyses are required to clarify the relationship with CNNM functions.

Our elemental analyses suggested that the *cnnm-1*; *cnnm-3* mutant worms had higher levels of Mg^2+^ in intestinal cells and lower levels of Mg^2+^ in the pseudocoelom, which contains other tissues such as the gonad (Table 1). This is consistent with the presumed molecular function of CNNM proteins at the basolateral membrane of the intestinal epithelia. In addition, we found that *cnnm-1*; *cnnm-3* mutants exhibited a severe gonadogenesis defect (Fig 4), which was completely restored by additional Mg^2+^ supplementation to the media. Collectively, these results strongly suggest that the gonadogenesis defect is due to Mg^2+^ deficiency in the gonad. Because of experimental limitations, we could only estimate magnesium levels in non-intestinal tissues, which showed moderate reduction in *cnnm-1*; *cnnm-3* mutant worms (~67% of wild-type worms). Whether this level of reduction alone can explain the pleiotropic phenotypes of mutant worms is unclear. However, a decrease of total magnesium levels by ~20% can cause proliferation arrest of HEK 293 human cultured cells [10]. More detailed studies of the Mg^2+^ distribution are required to precisely characterize the mechanism of how Mg^2+^ deficiency affects worm development.

The importance of Mg^2+^ in the regulation of various cell functions, such as proliferation, was predicted previously [42,43]. A study of chicken DT40 cells lacking TRPM7, a Mg^2+^-permeable cation channel, revealed the significance of Mg^2+^ influx in maintaining cell proliferation [44]. However, the mechanism of Mg^2+^ action is poorly understood. Our previous study showed that intracellular Mg^2+^ levels significantly affect ATP levels in cultured mammalian cells [13]. Because AMPK is an energy sensor kinase that is directly regulated by cellular ATP levels [39], AMPK was thought to play an important role in mediating the effect of Mg^2+^. In this study, we performed a genome-wide RNAi screen to identify genes involved in Mg^2+^-associated regulation of cell proliferation, which yielded 31 candidate genes including *aak-2*, which encodes a catalytic subunit of AMPK (Table 2). We confirmed the importance of AMPK by showing that additional mutations in *aak-1* and *aak-2* restored gonadal arm extension in *cnnm-1*; *cnnm-3* mutants (Fig 6). Moreover, in *cnnm-1*; *cnnm-3*; *aak-1*; *aak-2* quadruple mutant worms, fertility was restored at lower levels of Mg^2+^ supplementation (Fig 6). Therefore, we provide genetic evidence linking Mg^2+^ homeostasis to the AMPK function. In addition, tissue-specific RNAi experiments clearly located the rescue function of AMPK in somatic gonad (Fig 7). Therefore, Mg^2+^ decrease presumably causes gonadogenesis defect by affecting the AMPK function in somatic gonad, which then affects proliferation of germ cells.

One of the important downstream targets of AMPK is TORC1: activated AMPK suppresses the function of TORC1 [37]. Our RNAi experiments implicated *daf-15* (encoding Raptor ortholog) and *let-363* (encoding ortholog of mammalian TOR kinase) in the restored elongation of gonads by *aak-1*/*aak-2* mutation (S5 Fig), suggesting the importance of AMPK-TORC1 signaling in mediating gonadogenesis. It should be noted that Mg^2+^ influx through TRPM7 in chicken culture cells is essential for sustained activation of TORC1 and cell proliferation [44]. This Mg^2+^-dependent TORC1 signaling is presumed to be mediated by phosphoinositide 3-kinase and Akt. Whether it is functionally related to the Mg^2+^-dependent AMPK-TORC1 signaling in *C*. *elegans* remains unknown, but it is intriguing that Mg^2+^ perturbation convergently affects the same target molecule in distant species.

**Fig 8:**
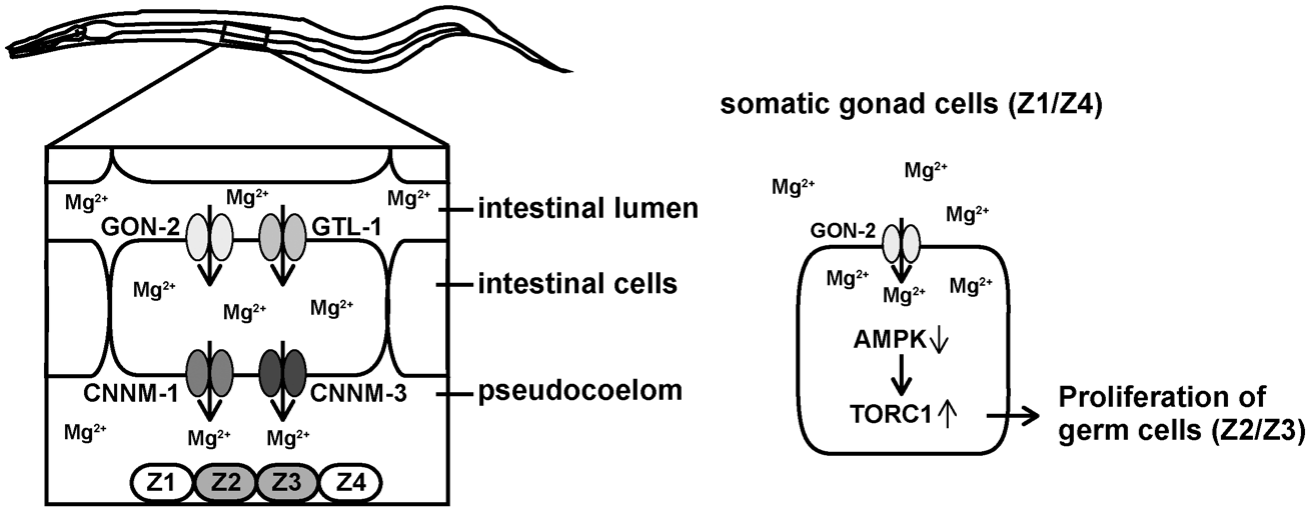
Proposed model for the role of *C*. *elegans* CNNM proteins. (Left) Mg^2+^ in the intestinal lumen enters into the intestinal cells through GON-2 and GTL-1, the apically-localized Mg^2+^-permeable channels. Mg^2+^ is then extruded from the intestinal cells to the pseudocoelom by CNNM-1 and CNNM-3, the Mg^2+^ transporters localized at the basolateral membrane. Pseudocoelom is in contact with many tissues/cells including primordial gonad cells (Z1−Z4). (Right) Through GON-2, somatic gonad cells (Z1/Z4) take up Mg^2+^, which suppresses the AMPK function and in turn augments the TORC1 function, ultimately stimulating the proliferation of adjacent germ cells (Z2/Z3).

Gonadal development in *C*. *elegans* is severely affected by the nutrition status, and starvation forces primordial gonad to stop cell proliferation due to the checkpoint activation. Reportedly, the starvation-induced proliferation arrest of germ cells can be partly rescued by *aak-1/2* mutation [45], as in the case of *cnnm-1*; *cnnm-3* mutant worms. Therefore, the arrest of germ cell proliferation in *cnnm-1*; *cnnm-3* mutants are presumed to occur by similar AMPK-dependent mechanism. In more detail, gonad cells are arrested at different stages of the cell cycle by starvation, depending on the cell types: Z1/Z4 cells (somatic gonad cells) are arrested at the G1 phase, while Z2/Z3 cells (germ cells) are arrested at the G2 phase [46–48]. It is not determined at which stage cells are arrested in *cnnm-1*; *cnnm-3* mutants, and thus, it is impossible to further evaluate the similarities between the starved worms and *cnnm-1*; *cnnm-3* mutant worms. However, it is reported that inactivation of GON-2 Mg^2+^ channel causes G1 arrest in Z1/Z4 cells by upregulating the G1/S checkpoint molecule CKI-1 [46]. Also, in chicken cell culture experiments, TRPM7-deficiency caused downregulation of TORC1 signaling and G1 arrest [44]. Taken together, it is plausible that Mg^2+^ shortage in the pseudocoelom of *cnnm-1*; *cnnm-3* mutant worms causes G1 arrest in Z1/Z4 cells by the AMPK-TORC1-dependent checkpoint control mechanism, and subsequently causes G2 arrest in Z2/Z3 cells (Fig 8).

Finally, besides *aak-2*, our RNAi screen yielded several other candidate genes, which are involved in various biochemical/biological processes. Further characterizations of the relationship between Mg^2+^ and these genes may reveal new roles for Mg^2+^ in the regulation of biological functions.

## Materials and Methods

### *C*. *elegans* strains and general methods for handling worms

All *C*. *elegans* strains used in this study were derived from wild-type *C*. *elegans* var. Bristol (N2). Unless otherwise indicated, the worms were grown at 20°C on NGM-lite plates seeded with OP-50 *E*. *coli*, as described previously [24]. The following mutations, rearrangement, and transgenes were used in this study: LGI *cnnm-4(dcr3)* and *daf-16(mgDf50)*; LGII *rrf-3(pk1426)*; LGIII *daf-2(e1370)*, *cnnm-5(ttTi19567)*, and *aak-1(tm1944)*; LGIV *cnnm-1(gk222902)* and *nT1[qIs51]* (IV; V); LGV *rde-1(ne219)*; LGX *cnnm-3(dcr2)*, *cnnm-2(dcr1)*, and *aak-2(ok524)*; *qIs56[lag-2p*::*GFP]*; *qIs140[lag-2p*::*rde-1]*; *sEx rCesC33D12*.*2*::*GFP 14584[rCesC33D12*.*2rCesC33D12*.*2*::*GFP*:: *GFP]*. Strains carrying each mutation and rearrangement were either isolated from the Trimethylpsoralen/UV-mutagenized library (see the “isolation of *cnnm* mutant strains” section for details), provided by the Caenorhabditis Genetics Center which is funded by NIH Office of Research Infrastructure Programs (P40 OD010440), or obtained from NEMAGENETAG Project funded by the European Community [49]. All isolated/obtained strains were backcrossed at least 4 times with N2 before use. Double homozygous worms for *cnnm-1(gk222902)* and *cnnm-3(dcr2)* are completely sterile, and therefore, *cnnm-1(gk222902)/+*; *cnnm-3(dcr2)* worms were maintained using the *nT1[qIs51]* (IV; V) balancer. Synchronization of worm development was achieved by egg laying of gravid adults for 6 h unless otherwise indicated.

### Generation of plasmids and transgenic *C*. *elegans* lines

To generate *cnnm-1p*::*GFP*, the 4,333-bp fragment of the 5′ region of *cnnm-1* was amplified by PCR and then inserted into the GFP expression vector pPD95.77 (kindly provided by A. Fire). To generate the *cnnm-1* genomic construct, the *cnnm-1* genomic fragment (−4,933 to +7,492 relative to the ATG start codon) was obtained by restriction enzyme digestion from the fosmid WRM0636cE07 (Dnaform). The remaining portion of the *cnnm-1* genomic fragment (+7,493 to +8,924) was generated by PCR amplification. For the *cnnm-3* genomic construct, the *cnnm-3* genomic fragment (−2,935 to +4,090) was obtained by PCR amplification. Thereafter, each genomic fragment was inserted into pBluescript KS (Stratagene). For the *cnnm-1*::*Venus* translational fusion construct, the same fosmid fragment as that used for the *cnnm-1* genomic construct was linked to the *cnnm-1* genomic fragment (+7,493 to +8,610) generated by PCR amplification. The fragments were then inserted into pPD95.79-venus (kindly provided by T. Ishihara). To express the venus-fusion protein of CNNM-3, the *cnnm-3* genomic fragment (−2,935 to −1) and the *cnnm-3* cDNA were both prepared by PCR, and these fragments were then inserted into pPD95.79-venus. To express *cnnm-1* or *cnnm-3* under the control of the *ges-1* promoter or *aex-3* promoter, *cnnm-1* or *cnnm-3* cDNA was prepared by RT-PCR and then inserted into pDEST-*ges-1p* and pDEST-*aex-3p*, kindly provided by H. Kuroyanagi [50,51]. To generate plasmids for feeding RNAi experiments (to target genes that are not included in the Ahringer library), cDNA fragments for *let-363* and *daf-15* were prepared by RT-PCR with the following primer sets: *let-363*; 5′-ACTAGTGCCGATAGACAGAACAAAGCAGCC-3′ and 5′-GTGGTACCGGACAAGCCATTCAACACCTTC-3′; *daf-15*; 5′-GTGCTAGCCCTCGTTTGCAGAACGTTTGAC-3′ and 5′-AGGTACCCCAGTTGAGCTCTCCGAGCACAG-3′. Amplified fragments were then inserted into L4440 (kindly provided by A. Fire). DNA fragments were inserted by conventional methods utilizing the restriction enzymes and ligases, with the exception of the expression construct for venus-fused CNNM-3, which was generated using the Gibson assembly method [52]. The DNA sequences of all PCR products were confirmed by sequencing. To generate transgenic lines, plasmids were injected into N2 or *cnnm-1(gk222902)/nT1[qIs51]*; *cnnm-3(dcr2)* along with *rol-6(su1006)* [53] or *rab-3p*::*mCherry* (Addgene) as an injection marker.

### Isolation of *cnnm* mutant strains

The mutant strains of *cnnm-2(dcr1)*, *cnnm-3(dcr2)*, and *cnnm-4(dcr3)* in this study were isolated from the Trimethylpsoralen/UV-mutagenized library by performing nested PCR as described previously [54]. The primer sets used for screening were as follows: *cnnm-2* first round; 5′-TGTCCCGTTTGATGGAAAAT-3′ and 5′-TTTGGAACTATCGTGCCTCC-3′; *cnnm-2* second round; 5′-CGAGGATGGTAGAAATGCTCA-3′ and 5′-TACCTGTGGCATCATGGTTG-3′. *cnnm-3* first round; 5′-TTGATTAGCGGCAATAAGGG-3′ and 5′-ATATGCCAAAATGGCTTTCG-3′; *cnnm-3* second round; 5′-GCTCACCATTCAACGATTCA-3′ and 5′-ATGAACTCACGAGGTGTCGG-3′. *cnnm-4* first round; 5′-CATTTTTCAGCGAGCCTTTC-3′ and 5′-CCCATCTTCTTCCGAATCAA-3′; *cnnm-4* second round; 5′-CTTTGCCTCGGTTTATCTGC-3′ and 5′-AGACGTGAATGGCCTTGTTC-3′. The *cnnm-1(gk222902)* and the *cnnm-5(ttTi19567)* alleles were generated by the *C*. *elegans* Reverse Genetics Core Facility at the University of British Columbia and the NEMAGENETAG Project, respectively.

### Germ cell counts

Germ cells were stained as previously described [55] with slight modifications. L4/young adults were permeabilized using the freeze–crack method and sequentially fixed in cold methanol for 10 min and in cold acetone for 10 min. The samples were blocked with 2% bovine serum albumin in PBS-T (PBS containing 0.05% Tween 20) for 30 min at room temperature, and then incubated overnight at 4°C with mouse anti-PGL-1 antibody K76 (1:20 dilution), developed by S. Strome [56] and provided by the Developmental Studies Hybridoma Bank. This was followed by incubation with Alexa Fluor 568 goat anti-mouse IgG (1:2000 dilution, Invitrogen) for 2 h at room temperature. Coverslips were mounted on a microscopic glass slide. Next, the samples were observed using a microscope, and PGL-1-positive cells were counted as germ cells.

### Gonadal arm extension

To analyze gonadal arm extension, *lag-2p*::*GFP* was used to visualize the Z1/Z4 cells and DTCs. The worms were analyzed at the L4/young adult stage, unless otherwise noted, using a microscope. Based on visual observation of the *lag-2p*::*GFP* signals and the phase contrast view, we determined the outline of the gonad, and worms with gonads of ≥ 60 μm in length of the long axis (3-fold length of the primordial gonad) were defined as extended.

### Lifespan assay

A lifespan assay was performed as described previously [20] with slight modifications, starting with L4/young adults. To remove contamination with progeny, worms were transferred to fresh NGM-lite plates seeded with OP-50 every 2 days until day 8, after which only those worms on plates where progeny was observed were transferred. Survival was monitored daily. Worms that did not move, respond to nose touch with a platinum picker, or exhibit pumping were determined as dead and were removed. Worms that crawled off the plate, had a protruded vulva, or died by internal hatching were excluded.

### Body size measurement

Mixed stage worms from L2 to adult (L4 to adult worms were most abundant) were anesthetized with M9 buffer containing 50 mM NaN_3_ and were mounted on a 3% agarose pad on a microscopic glass slide. Thereafter, the worms were examined for alae formation using a microscope and then photographed for body size measurement. The area of the worms was directly measured from the images using Image J (NIH software). The body size at the transition from L4 to adult molt was determined by calculating the mean body size of the smallest three worms with alae and the largest three worms without alae.

### Male tail observation

Observation of the male tail was performed as previously described [57]. Adult worms were anesthetized with 10 mM sodium azide and transferred to a 5% agar pad. The worms were turned over with a pick to the ventral side up and immediately covered with a coverslip.

### Genome wide RNAi screening

Feeding RNAi was performed as described previously [58]. In total, 15,357 bacterial RNAi feeding strains from the Ahringer library [29] were tested as follows in the first round screening (*n* = 5–10 worms per strain), using bacteria carrying the empty vector L4440 as the negative control. *cnnm-1(gk222902)/nT1[qIs51]*; *cnnm-3(dcr2)*; *rrf-3(pk1426)*; *qIs56[lag-2p*::*GFP]* gravid adults were bleached, and synchronized P0 worms at the L1 stage were transferred to RNAi plates. F1 progeny lacking the balancer *nT1[qIs51]* (without pharyngeal GFP expression) were phenotypically scored at the L4/young adult stage as described in the “Gonadal arm extension” section. The wells were scored as positive if gonadal arms extended in more than 50% of worms in the F1 generation. Some wells showed larval arrest or sterile phenotypes in the P0 generation, and therefore, these wells were scored in the P0 generation. The first round screening led to the identification of 119 positive wells, which was followed by sequence analyses to identify the RNAi clone in each well. Because some wells contained multiple clones, we re-transformed each of the 135 sequence-verified clones into the HT115 *E*. *coli* strain and performed the second round screening as described above (*n* = 30 worms per clone).

### ICP-MS

To measure magnesium levels in whole worms, 300 synchronized L4/young adult worms were incubated for 30 min with washing buffer containing 110 mM HNO_3_ (semiconductor grade, Wako) and 187 mM NH_3_ (ultrapure grade, Kanto Chemical), which corresponds to approximately 300 mOsm/l and pH 7.0–8.0 at room temperature, and were then washed 5 times with washing buffer. Subsequently, worms were boiled at 95°C for 5 min and sonicated using Bioruptor (UCD-250HSA; Cosmo Bio). The homogenates were completely dried by incubation at 98°C, and then subjected to treatment with 100 μl of 40% HNO_3_ at 95°C for 2 h. The solution was diluted to 1 ml with ddH_2_O and magnesium levels were determined using ICP-MS (7700x; Agilent), according to the manufacturer’s instructions. The magnesium levels were normalized to total protein levels, which were determined using the BCA assay kit (Thermo Scientific). A blank sample was prepared using the same procedure without worms. To measure magnesium levels in the intestine, approximately 300 synchronized L4/young adult worms were cut with a scalpel just behind the pharynx in a drop of washing buffer. The extruded intestine was cut away from the remnants of the body, and the isolated intestines were then washed twice with washing buffer. Magnesium levels were analyzed as described above.

### Volume calculation

Body volume was measured as described previously [59,60] with slight modifications. L4/young adults were transferred to M9 buffer containing 50 mM NaN_3_ on coverslips and then photographed. The total body volume of the worms was calculated by assuming that the body shape was composed of two cones (from the tip of the nose to the anterior end of the intestine, and from the posterior end of the intestine to the tip of the tail) and a cylinder (remaining body part) and by measuring each length and radius. Intestine volume was calculated by assuming that the shape was cylindrical.

### Microscopy

Fluorescence images were acquired using an inverted microscope (IX81; Olympus) equipped with a laser scanning confocal imaging system (FluoView FV1000; Olympus). Nomarski images were collected concurrently or alone using the same microscope using Nomarski optics. A multiline argon laser and image analysis system (FV10-ASW; Olympus) were also used for image acquisition. To analyze gonadal arm extension, *lag-2p*::*GFP* fluorescence was observed using a stereo microscope (SZX7; Olympus) equipped with a U-RFL-T 100W mercury lamp (U-RFL-T; Olympus).

### Statistics

All statistical analyses were performed using GraphPad Prism 6 software (GraphPad Software) and are presented as the mean ± SEM. *p* values were obtained by Student’s two-tailed *t*-test for Table 1 and by ANOVA, followed by two-tailed multiple Student’s *t*-test with Tukey’s correction for Figs 2C and 3B. For lifespan assays (Figs 2D, 3C and S2 Fig), we used the log rank (Mantel-Cox) test. Bonferroni correction was applied to multiple comparisons of lifespans.

## Supporting Information

S1 Fig Alignment of amino acid sequences of *C*. *elegans* CNNM family proteins. Asterisk (*) and colon (:) denote identical residues and conserved substitutions, respectively. The sequence of *H*. *sapiens* CNNM4 is also shown. The regions for functionally essential DUF21 and CBS domains, and the amino acids for which coding nucleotide sequences were directly lost/changed by each genetic alteration, are highlighted. *cnnm-1*(*gk222902)* contains a point mutation that led to a premature stop codon in place of arginine residue 255 in the DUF21 domain. *cnnm-2(dcr1)* contains a deletion of 162 nucleotides from the inside of exon 11 to the inside of exon 12, leading to the loss of 40 amino acids in the CBS domain. *cnnm-3(dcr2)* contains a deletion of 289 nucleotides that include the splice acceptor site of intron 4 and 183 nucleotides in the following exon 5. This deletes 61 amino acids in the DUF21 domain and causes additional deletion/alterations because of incorrect splicing. *cnnm-4(dcr3)* contains a deletion of 173 nucleotides that include the entire exon 5 and the splice acceptor and donor sites in adjacent introns. This deletes exon 5-encoded 41 amino acids in the DUF21 domain, and causes frameshifts if RNA splicing occurs by directly linking exon 4 and exon 6. *cnnm-5(ttTi19567)* contains the *Mos 1* sequence inserted in exon 2, resulting in a truncated product that lacks both the DUF21 and CBS domains.
(TIF)

S2 Fig Overexpression of CNNM can extend lifespan. For each genotype, 45 synchronized L4/young adult worms were transferred to fresh plates (15 worms per plate) and then scored daily for survival. The graph represents data combined from at least two experiments. Mean lifespan (± SEM) of worms is also indicated in parentheses. *p* values were determined by log rank (Mantel-Cox) test, and the Bonferroni method was then used to correct for multiple comparisons. ****p < 0.0001.
(TIF)

S3 Fig *cnnm-1*; *cnnm-3* mutant male worms show normal tail ray development. Nomarski images of the ventral view of adult wild-type and *cnnm-1*; *cnnm-3* mutant mail tails. The anterior side of the worm is positioned to the left. Bar, 20 μm.
(TIF)

S4 Fig Representative images showing the effect of RNAi screening. Representative images of L4/young adult worms of *cnnm-1*; *cnnm-3*; *rrf-3* mutants with *Ex[lag-2p*::*GFP]*, which were fed with bacteria carrying the dsRNA corresponding to the indicated genes (the results of the top 5 genes in second round screening are shown). In each image, the anterior side of the worm is positioned to the left. Dotted lines indicate the outlines of the gonad. As a negative control, the worms were fed with bacteria carrying the empty vector L4440. Bar, 20 μm.
(TIF)

S5 Fig Importance of TORC1 signaling. *cnnm-1*; *cnnm-3*; *aak-1*; *aak-2* mutant worms, carrying *lag-2p*::*GFP*, were fed with bacteria carrying the dsRNA corresponding to the indicated genes. Because RNAi of either *let-363* or *daf-15* is known to cause L3 larval arrest [38], we observed the worms at the L2–L3 stages. In each image, the anterior side of the worm is positioned to the left. Dotted lines indicate the outlines of the gonad. Bar, 20 μm. Worms with gonads of ≥ 60 μm in length of the long axis (3-fold as long as the primordial gonad) were determined as extended and the results (%) are shown in the right graph. More than 50 worms were analyzed for each condition.
(TIF)

S1 Dataset Numerical data for figures and tables. The underlying numerical data for each figure or table are shown in separate sheets.
(XLSX)
