# Supplementary figures and images for: Mg2+ Extrusion from Intestinal Epithelia by CNNM Proteins Is Essential for Gonadogenesis via AMPK-TORC1 Signaling in Caenorhabditis elegans
Source: PLoS Genet. 2016 Aug 26;12(8):e1006276. doi: 10.1371/journal.pgen.1006276 (PMC5001713; doi:10.1371/journal.pgen.1006276)

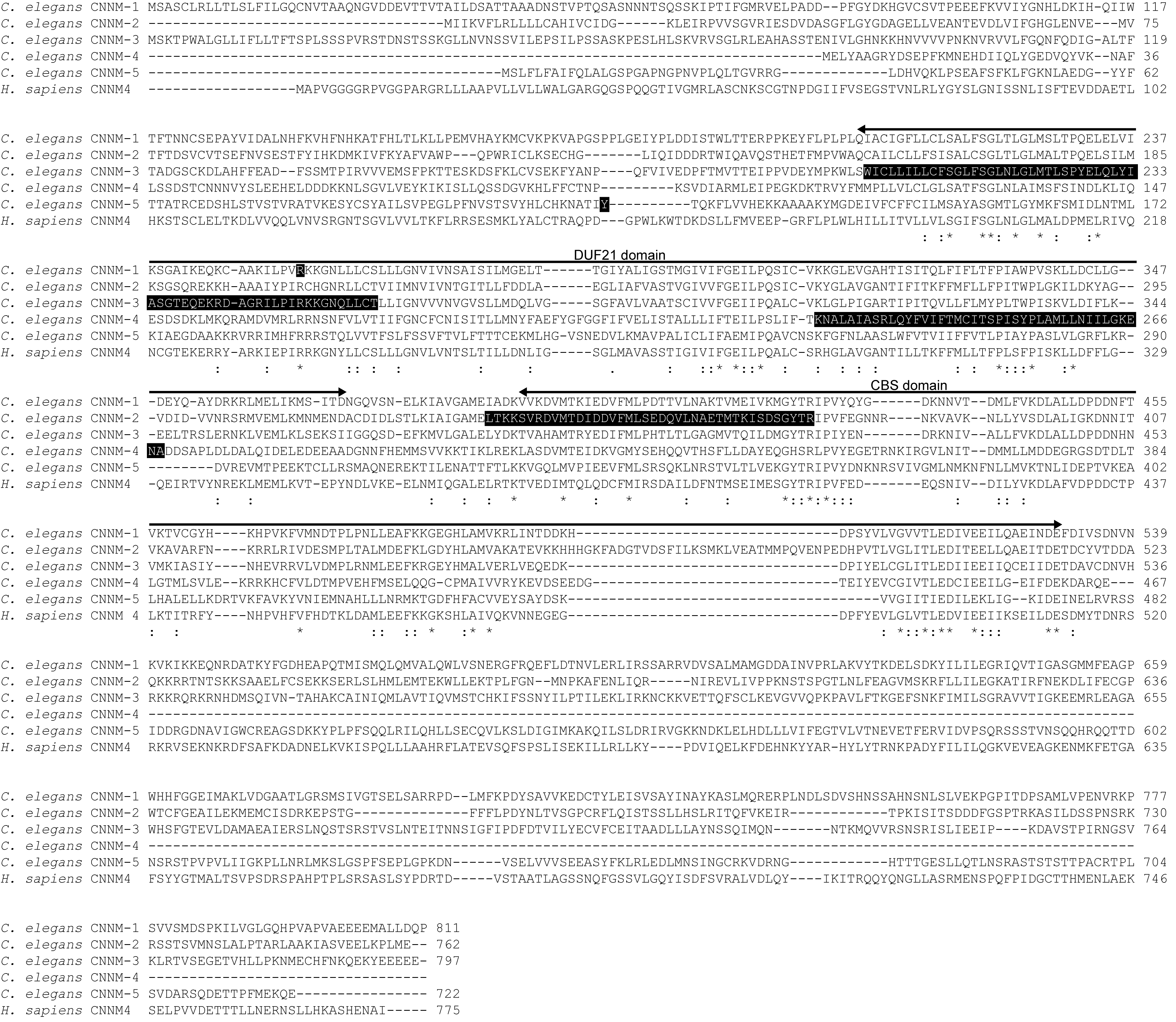

Supplement: S1 Fig — Asterisk (*) and colon (:) denote identical residues and conserved substitutions, respectively. The sequence of H. sapiens CNNM4 is also shown. The regions for functionally essential DUF21 and CBS domains, and the amino acids for which coding nucleotide sequences were directly lost/changed by each genetic alteration, are highlighted. cnnm-1(gk222902) contains a point mutation that led to a premature stop codon in place of arginine residue 255 in the DUF21 domain. cnnm-2(dcr1) contains a deletion of 162 nucleotides from the inside of exon 11 to the inside of exon 12, leading to the loss of 40 amino acids in the CBS domain. cnnm-3(dcr2) contains a deletion of 289 nucleotides that include the splice acceptor site of intron 4 and 183 nucleotides in the following exon 5. This deletes 61 amino acids in the DUF21 domain and causes additional deletion/alterations because of incorrect splicing. cnnm-4(dcr3) contains a deletion of 173 nucleotides that include the entire exon 5 and the splice acceptor and donor sites in adjacent introns. This deletes exon 5-encoded 41 amino acids in the DUF21 domain, and causes frameshifts if RNA splicing occurs by directly linking exon 4 and exon 6. cnnm-5(ttTi19567) contains the Mos 1 sequence inserted in exon 2, resulting in a truncated product that lacks both the DUF21 and CBS domains. (TIF) [file pgen.1006276.s001.tif]

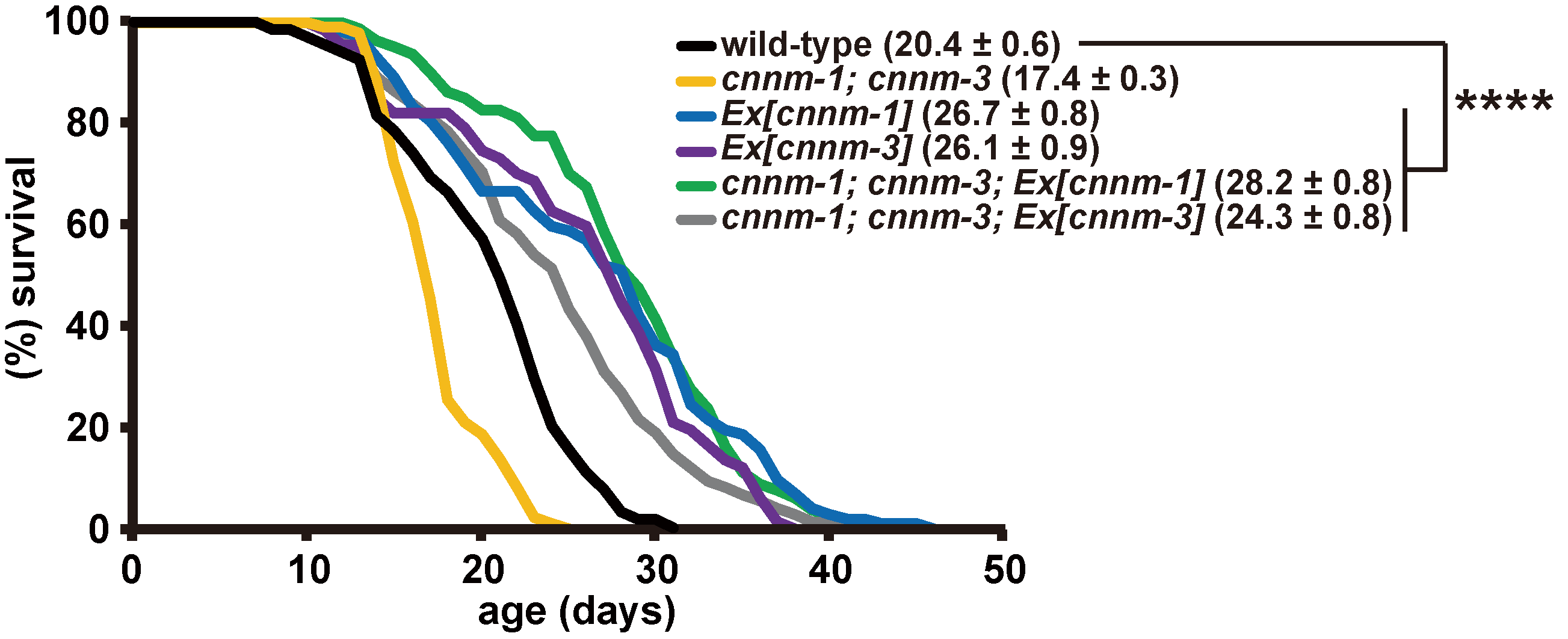

Supplement: S2 Fig — For each genotype, 45 synchronized L4/young adult worms were transferred to fresh plates (15 worms per plate) and then scored daily for survival. The graph represents data combined from at least two experiments. Mean lifespan (± SEM) of worms is also indicated in parentheses. p values were determined by log rank (Mantel-Cox) test, and the Bonferroni method was then used to correct for multiple comparisons. ****p < 0.0001. (TIF) [file pgen.1006276.s002.tif]

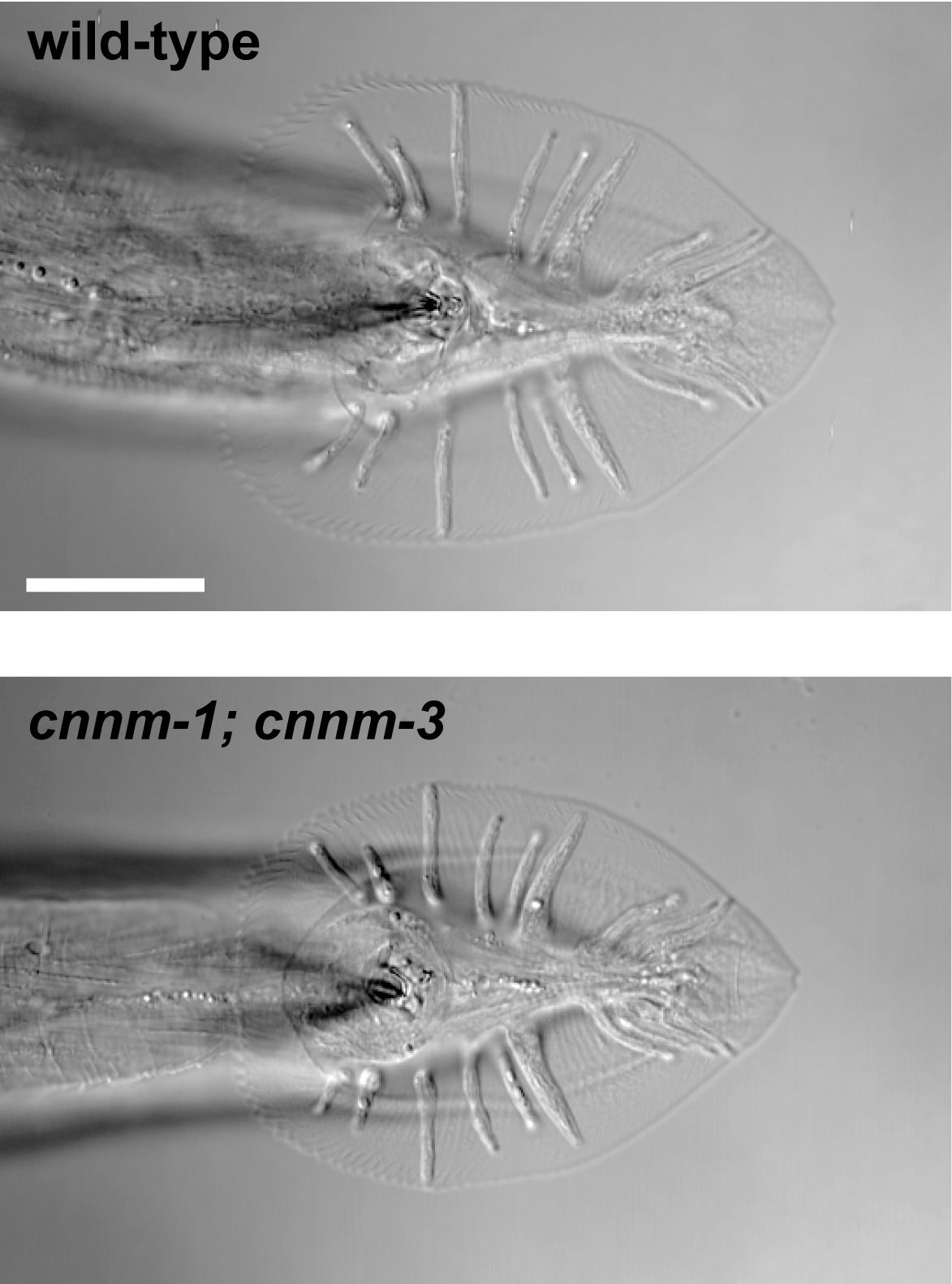

Supplement: S3 Fig — Nomarski images of the ventral view of adult wild-type and cnnm-1; cnnm-3 mutant mail tails. The anterior side of the worm is positioned to the left. Bar, 20 μm. (TIF) [file pgen.1006276.s003.tif]

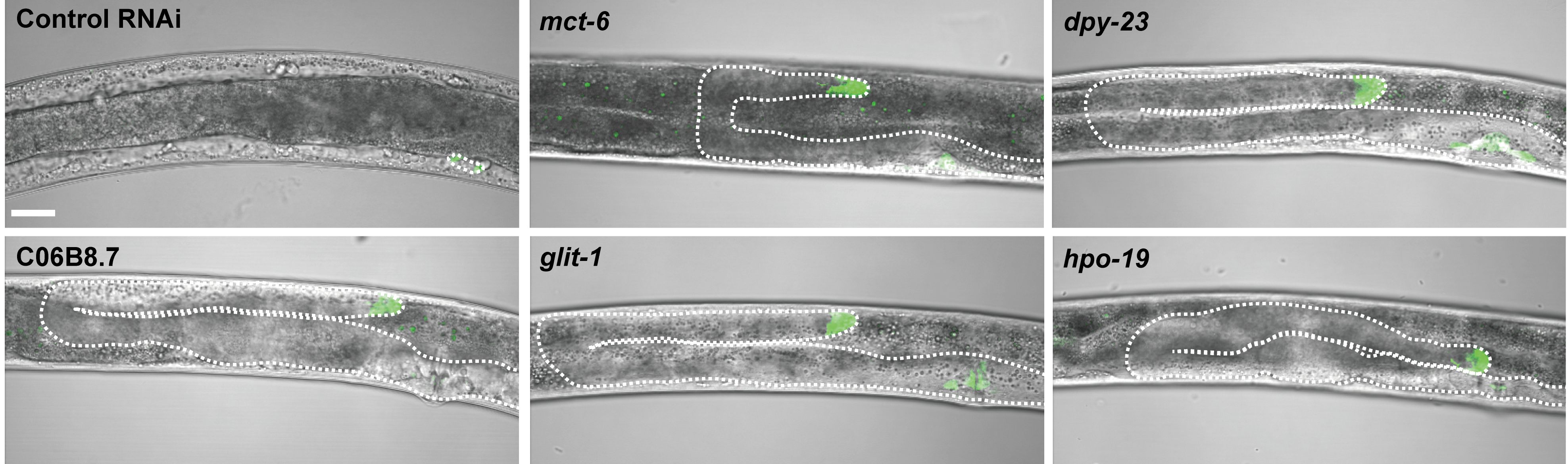

Supplement: S4 Fig — Representative images of L4/young adult worms of cnnm-1; cnnm-3; rrf-3 mutants with Ex[lag-2p::GFP], which were fed with bacteria carrying the dsRNA corresponding to the indicated genes (the results of the top 5 genes in second round screening are shown). In each image, the anterior side of the worm is positioned to the left. Dotted lines indicate the outlines of the gonad. As a negative control, the worms were fed with bacteria carrying the empty vector L4440. Bar, 20 μm. (TIF) [file pgen.1006276.s004.tif]

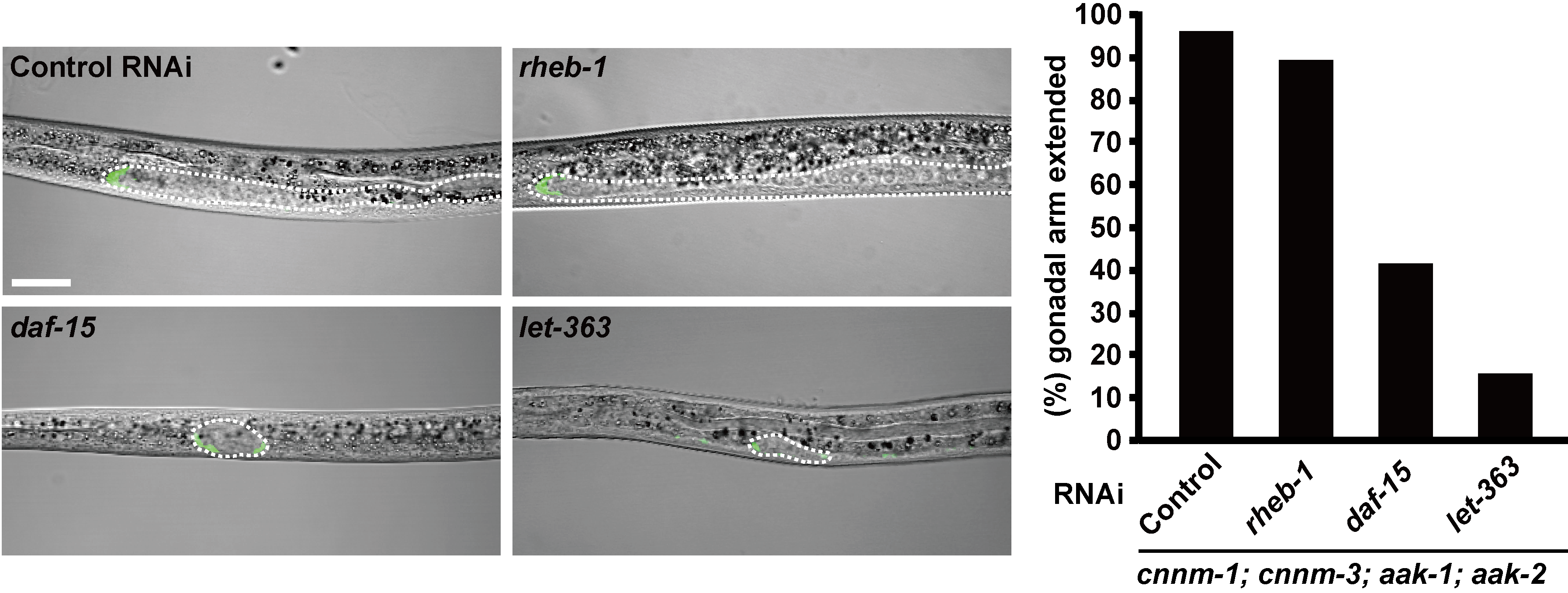

Supplement: S5 Fig — cnnm-1; cnnm-3; aak-1; aak-2 mutant worms, carrying lag-2p::GFP, were fed with bacteria carrying the dsRNA corresponding to the indicated genes. Because RNAi of either let-363 or daf-15 is known to cause L3 larval arrest [38], we observed the worms at the L2–L3 stages. In each image, the anterior side of the worm is positioned to the left. Dotted lines indicate the outlines of the gonad. Bar, 20 μm. Worms with gonads of ≥ 60 μm in length of the long axis (3-fold as long as the primordial gonad) were determined as extended and the results (%) are shown in the right graph. More than 50 worms were analyzed for each condition. (TIF) [file pgen.1006276.s005.tif]
